# A Pilot Study of the Impact of NHS Patient Transportation on Older People with Dementia

**DOI:** 10.4061/2010/348065

**Published:** 2010-06-14

**Authors:** Nicola Roberts, Stephen Curran, Virginia Minogue, Jane Shewan, Rebecca Spencer, John Wattis

**Affiliations:** ^1^South West Yorkshire Partnerships NHS FoundationTrust, Block 9, Fieldhead Hospital, Ouchthorpe Lane, Wakefield WF1 3SP, UK; ^2^University of Huddersfield, Old Age Psychiatry, South West Yorkshire Partnership NHS Foundation Trust, Calder Unit, Fieldhead Hospital, Ouchthorpe Lane, Wakefield WF1 3SP, UK; ^3^Research & Development, West Yorkshire Mental Health R & D Consortium, Leeds Partnership Foundation NHS Trust, St. Mary's House, St. Mary's Road, Leeds LS7 3JX, UK; ^4^Research & Effectiveness, The Mid Yorkshire Hospitals NHS Trust, Rowan House, Pinderfields General Hospital, Aberford Road, Wakefield WF1 4EE, UK; ^5^Old Age Psychiatry, Harold Wilson Building, The University of Huddersfield, Queensgate, Huddersfield HD1 3DH, UK

## Abstract

*Background*. A pilot study using a mixed methodology was used to evaluate the effects of travelling on NHS Patient Transport Service ambulances on the experience of patients with dementia. The study assessed the feasibility of using Dementia Care Mapping in this setting and looked at the effects of the presence of designated staff teams on journeys, compared to journeys without such designated staff. *Method*. Dementia Care Mapping was used to observe and record participants' behaviour, mood, and engagement during their outward and return journeys to NHS hospital sites. Observations were analysed for themes relating to the effects of travelling on PTS across the two groups. *Results and Conclusions*. Participant's observed mood scores did not differ significantly across the two groups but the range of behaviours recorded on the escorted group journeys did and were reflective of formal care environments. The findings from this study highlight the importance of trained escorts on NHS PTS ambulances for people with dementia and provide important information regarding further research in this area.

## 1. Introduction

Nonemergency Patient Transportation Services (PTS) accounts for over 80% of all patient journeys by ambulance in England but despite its importance in healthcare delivery, a review by the Audit Commission [1] reported that nonemergency PTS often has a much lower profile than emergency ambulance services and within the NHS as a whole. In terms of allocating resources, nonemergency PTS receives a declining proportion of funds; currently only 20% of total ambulance service expenditure are spent on PTS, compared to over 25% in the 1990s.

The key findings in this review [1] were that service users reported concerns about the overall length of the patient day, the wait to be seen, and the delays before being taken home. Time spent in the vehicle, with lack of information about how long people will have to wait to travel or spend in vehicles, was also an area of concern for service users. The review [1] recommended that transport arrangements should be treated as central to access to services and as part of the package of healthcare, and nonemergency PTS should be treated as a core part of the NHS.

Another report describes the elements of a patient-led NHS and the importance of the emotional experience of patients [2]. The 5 dimensions of the patient experience include “access and waiting,” which covers the extent to which patients are able to reach required services and treatments when needed, waiting times, and patients' ability to physically get to services [2]. This has clear implications for PTS as they represent part of the healthcare package, given that they are the first contact with NHS services that the patient will encounter on each occasion, and therefore have a responsibility to enhance the patient experience.

Research has shown that patient transportation is vital, particularly for service users who are vulnerable or isolated [3]. Older adults and people with dementia often do not have access to private transport and can no longer use public transport for fear of getting lost [4]. The elderly are often very reliant on patient transport services and people suffering from dementia are even more so. As the disease progresses, most people with dementia will eventually lose their ability to drive and, unless they are supported to access transport services, they will become increasingly isolated [5]. Many dementia service users need to regularly attend day clinics or memory clinics for monitoring and assessments and increasingly, their only method of transport is to use NHS patient transport services. Some evidence suggests that this can be a negative and unsettling experience for them [6], with people being left waiting, being taken to the wrong place and being upset by other people using the same transport. 

No research to date has been conducted on the impact of PTS on people with dementia as eliciting the views and experiences of dementia sufferers has historically been very problematic. However, Dementia Care Mapping (DCM) is an observational method developed by Kitwood and Bredin [7] to assess quality of life and care from the perspective of the person with dementia in formal care settings such as care homes, day hospitals, wards, and day centers. This tool is rooted in the philosophy of person-centred care, which endorses the “personhood” of people with dementia. Tom Kitwood explained that DCM is “a serious attempt to take the standpoint of the person with dementia, using a combination of empathy and observational skill” ([7], page 4: in [3]). 

There has been much research conducted using DCM to assess the quality of life and care for people with dementia in a variety of settings; a review of the research literature showed that DCM has been applied in nursing homes, hospital facilities, day care facilities, long-term care, and assisted living facilities with great success [8]. Some studies have applied the use of DCM in nondementia settings such as geriatric hospital wards [9] and in learning disability services [10]; however, there has been no DCM research undertaken to date which looked at its feasibility on PTS ambulances for people with dementia. Given the importance that this necessary journey has on the well-being of people with dementia, this study could assist with identifying how to promote safety and reduce stress during the transportation of people with dementia. The findings could also help to inform the planning and modernisation of current services and may provide support for more community-based models of care and support for this important patient group.

This pilot study evaluated the impact of patient transportation from home to day clinic on the well-being of people with dementia. In line with recommendations from the Audit Commission [1], this study assessed the quality of care provided by nonemergency Patient Transport Services and the effects on patients' quality of life from the perspective of service users with dementia. This area had not been subject to research before so this study will help to determine the quality of services for people with dementia across several sites in West Yorkshire and Newcastle. The study used Dementia Care Mapping as the observational method to assess its feasibility in this setting as this had also not been subjected to previous research. This study was a multi-site project that included both urban and rural areas and different ethnic/demographic populations.

**Research Questions**

1. How feasible is the use of Dementia Care Mapping 8 [11] to monitor the care and experience of people with dementia on PTS ambulances in practice, and in research studies?
2. What are the observed behaviours and well-being of service users with dementia during a typical patient journey on PTS?
3. How does having designated PTS staff teams, with an escort, affect the well-being (NB: Definition of “well-being”: reflects person-centred care which is “a standing or status that is bestowed upon one human being, by others, in the context of relationship and social being. It implies recognition, respect and trust.” (Kitwood, 1997, page 8: in Brooker, 2005).) of people with dementia in comparison to generic PTS staff?

## 2. Method

### 2.1. Design

Using both a qualitative phenomenological perspective and a quantitative analysis of the observation data within a mixed methodology design, participants with dementia were observed and events recorded and analysed using Dementia Care Mapping (version 8), during their journey to a day/memory clinic. The process was then repeated for their journey home.

### 2.2. Service User and Carer Involvement

Service users and carers were involved in this pilot study from the beginning. Consultation took place at the development stage through service user and carer representation on the Ageing and Mental Health Research Group at the University of Huddersfield. Further consultation was conducted through service user and carer research and reference groups across the NHS Trust localities.

### 2.3. Setting

Participants were mapped from their home and throughout their journey on Patient Transport Services (PTS) to their destination (memory/dementia clinic), and for an hour after arrival, to evaluate the effects of transportation. The same participants were then mapped for an hour before they were due to be collected for their return journey, throughout their journey home on patient transport services until arrival at home. The participants use nonemergency PTS provided by their local Ambulance Trust.

### 2.4. Sample and Participants

A nonprobabilistic purposive sample of 13 service users was recruited for this study. All participants had a medical diagnosis of dementia or dementia-related disorder and were identified through NHS-provided memory or dementia clinics. The sample was taken from clinical populations in South West Yorkshire Partnership NHS Foundation Trust (SWPFT), Mid-Yorkshire NHS Trust, Newcastle, North Tyneside and Northumberland Mental Health NHS Trust and Craven, Harrogate & Rural District PCT (Airedale Hospital). The ambulance services that were mapped in this study were the Yorkshire Ambulance Service (YAS) and North East Ambulance Service (NEAS). Due to the confined environment of PTS ambulances, only 2-3 participants on each journey from each Memory Clinic/Dementia Service were invited to participate in this study.

**Inclusion Criteria**

It includes medical diagnosis of dementia or dementia-related disorder, Older adults, and early onset dementia.

**Exclusion Criteria**

It includes learning disabilities, significant distressing behavioural or psychological symptoms, and risk to self or others.

### 2.5. Ethics/Capacity to Consent

Service users with dementia or dementia-related disorder may not have been able to give their informed consent to participate in this study. To address this, the study sought professional opinion with regard to the person's last known level of cognitive functioning on formal testing to determine the most appropriate approach to gaining informed consent; for mild cognitive impairment, informed consent was sought from the individual while for more significantly impaired cognition, a letter and patient information sheet explaining the purpose and procedures of the study was sent to carers of the person with dementia, to seek their informed assent to the service user's participation in this study. 

Throughout the mapping sessions, on-going observation and consideration of the participants' well-being determined the continuation of informed assent; if an individual displayed behaviour that indicated distress or anxiety about being mapped, the consent/assent would have been taken as withdrawn and the mapping would have discontinued immediately. However, this was not observed during the data collection and all participants were able to be included in the study. 

These measures are in line with the guidance outlined within the Mental Capacity Act [12] for research. Although this study was undertaken before the requirements of the Act became law, the consent protocol for this study was peer reviewed by an Ethics Committee who judged it good practice. The consent protocol was rereviewed in light of the impending guidance and no changes were deemed necessary.

### 2.6. Measures

The observational tool of Dementia Care Mapping, version 8 (DCM 8) as described by Kitwood and Bredin [7] was used in this study. DCM 8 was developed to observe, record, and analyse the quality of life of people with dementia when receiving care within formal care environments. The mapping takes place in communal areas of care environments; after each 5-minute time period, two different codes are recorded that reflect what has happened to each individual being mapped. The two codes recorded in this process reveal two dimensions of patient experience: (1) the Behaviour Category Codes (BCC) describe 1 of 23 different participant behaviors (see Table 1) and (2) a Mood and Engagement value (ME) reflects an observation about the participant's mood and their level of engagement with the associated behaviour during each 5-minute period (see Table 2). Scores from the 15 journey maps were collated and analysed for qualitative data relating to BCC's and ME values. 

Behaviour Category Codes (BCC) are grouped into “high,” “moderate”, and “low” potential categories. These reflect the extent to which each category of behaviour can potentially provide positive mood and engagement for the service user, for example, being “Unresponded to” (U) will always reflect a low associated mood and engagement value.

Mood and Engagement (ME) values are always coded in the context of the BCC that they accompany. ME values range from extreme ill-being to extreme well-being and values are averaged to arrive at an overall ME score which provides an index of relative mood and engagement, in the associated BCC, for a specific time period. Also recorded are Personal Detractions (PD's) and Personal Enhancers (PE's) which are staff behaviours that can potentially impact on the “personhood” of the person with dementia. Observational criteria of the codes and their related values are operationally defined in the DCM manual to further enrich the reliability of data.

Mappers are required to demonstrate high concordance of interrater reliability with other mappers prior to conducting a DCM evaluation, to ensure that results are comparable. Mappers must achieve at least 70% concordance for evaluation purposes and at least 80% for research purposes; excellent inter-rater reliability (kappa value >  .8) has been demonstrated in previous studies [13]. For the purpose of this study, all mappers had to demonstrate at least 80% concordance prior to the PTS evaluation taking place.

### 2.7. Procedures

A research steering group was formed to oversee the project's coordination and progress. This group included the lead researcher and members of the mapping team, with representatives from the NHS Trust R & D departments, the local Ambulance NHS Trust, older peoples' services, and service users and carers.

Memory clinics and Dementia day clinics were identified within each of the Trust localities; the individual opening times of each service within each Trust informed the mapping schedule for that Trust. Each service was asked to assist in recruiting suitable participants for the study, based on the inclusion/exclusion criteria and leaflets, patient information sheets, and consent forms were then distributed to potential participants and their carers. Following informed consent/assent, 2-3 participants were selected from each service for inclusion in the study. 

A preliminary pilot study was carried out in Dewsbury prior to the main data collection using this protocol. Findings demonstrated that the protocol was robust and this informed the procedure for the remaining localities in the study.

Once each PTS journey to be mapped was identified, staff briefing sessions were carried out to inform staff and managers of the services and ambulances about the aims and objectives of the study. The research team attempted to dispel any fears or anxiety about the study to the PTS staff so that a true picture of service impact on participants was presented at the time of data collection. A team of trained Dementia Care Mappers, who had previously achieved at least 80% inter-rater reliability, was recruited to participate in the delivery of this study. To reduce observer effects, one mapper was allocated to each patient journey to map the participants while another trained mapper waited at the clinic to assist in the mapping procedure once the participants had arrived at the service.

For the return journey, two mappers observed and recorded participants' behaviour in the day clinic for an hour before the participants' were due to be collected. One mapper then undertook the mapping for the journey home for the rest of the observation period. Following each journey map, a short staff debrief was carried out with the transportation staff that were involved. Following completion of the data collection, the lead researcher collated all map scores and analysed them for results.

### 2.8. Recruitment Issues

The recruitment of participants to this study was quite problematic for a number of reasons. The main issue was around capacity to consent in that very often access to the carers or family members of suitable patients was limited. Even when access to carers/family was achieved and the patient had been successfully recruited, the mapper to be included on the ambulance journey was arranged in advance, often in distant locations, to find that on the day the patient's journey had had to be cancelled due to ill-health. These factors were impossible to anticipate and would mean that a great deal of preparation and coordination was lost. Another issue was the infrequency of patient visits to attend assessment appointments. This study found it difficult to recruit patients attending outpatients or memory clinics and would recommend that future research concentrates on patients attending day hospitals or care units for the elderly, where regular journeys are made on a weekly basis generally on the same day of the week.

## 3. Results

A total of 8 PTS services were mapped with all but one being mapped on both the outward and return journey giving 15 separate journey maps. Thirteen participants were recruited and observed for this study. The “typical” participant is a 75-year-old female with moderate dementia, travelling to a day hospital and having a journey time of 38 minutes. Participant demographic details and journey information are provided in Table 3. 

### 3.1. Behaviour Category Codes (BCC)

**Figure 1:**
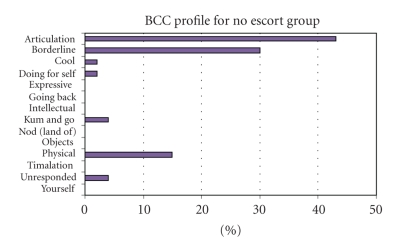
Behaviour category code profile—no escort group.

**Figure 2:**
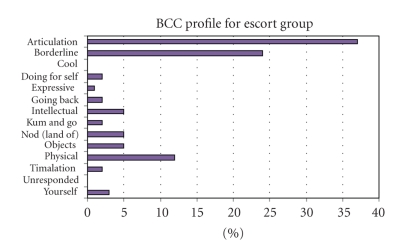
Behaviour category code profile—escort group.

The analysis looked at the BCC profiles of the two groups for a comparison of the types of behaviours observed on PTS, with and without a designated team and escort; the key for all BCC's can be seen in Table 1. The percentage of time that patients spent in particular behaviour categories while travelling on PTS across the two groups can be seen in Figure 1 (no-escort group) and Figure 2 (escort group).

From these figures, it is apparent that the escort group experienced a much wider range of positive behaviours than the no escort group whose experiences were mainly limited to the behaviours of “Articulation” 43%, “Borderline” 30%, and “Physical” 15%.

The observed behaviour categories of “Cool” 2%, “Unresponded to” 4%, and “Borderline” 30% in this group are all low and moderate potential categories, respectively, meaning that they have limited potential for positive mood and engagement. None are reflective of person-centred care and it is recommended that patients are brought up to a high potential category as soon as possible [11].

In contrast, the range of behaviours observed in the escort group (Figure 2) reflects the presence of trained PTS escorts who are often familiar to the patients through regular journeys. The most common occurrences of BCC's in the escorted group were seen as “Articulation” 37%, “Borderline” 24%, and “Physical” 12%; however a much wider range of positive behaviours was recorded that would normally be observed in formal care environments, for example, “Expressive” 1%, “Going back” 2%, and “Intellectual” 5%.

In the escort group, all behaviours observed were high potential categories, except for “Borderline” which is classed as moderate. However, the percentage of time that patients spent in B was lower in the escorted group (24%) than in the nonescorted group (30%). All other behaviours observed in the escort group reflected person-centred care, as defined by the DCM manual [11] and were supportive of the patients with dementia.

### 3.2. Mood and Engagement (ME) Values

**Figure 3:**
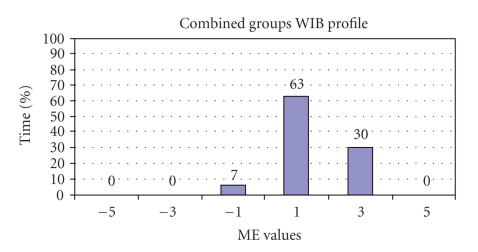
Group ME profile—Escort Group.

**Figure 4:**
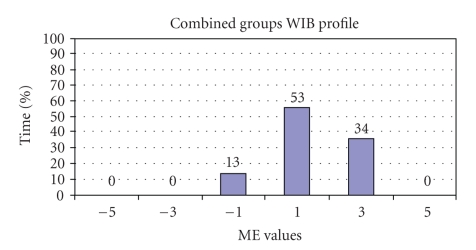
Group ME profile—No Escort group.

Group Mood and Engagement, previously known as well/ill being (WIB), values were also compared between having a dedicated PTS team with an escort to having generic PTS services, to determine the percentage of time that the two groups spent in different ME values; this shows the impact of having a known PTS staff team (driver and escort) on the well-being of patients with dementia in comparison to generic PTS drivers. Figure 3 shows the ME profile for the “escort” group (*n* = 8) while Figure 4 shows the ME profile for the “no escort” group (*n* = 5).

A Mann Whitney *U* test was used to compare the level of well-being observed in each of the two groups. The results were not significant (*U* = 16, *P* = .56) suggesting that in this sample, travelling with a known PTS staff team with an escort was not a significant factor in patients' mood and level of engagement.

A slightly higher percentage of time spent in +3 (Content, happy, relaxed, considerable positive mood) was seen in the no escort group which was not expected. Yet the profile for −1 and +1 (Neutral, no overt signs of negative or positive mood) did differ, showing less −1 values in the escort group (7%) to the no escort group (13%) and higher +1 values in the escort group (63%) to the no escort group (53%). These results suggest that the presence of a known escort is related to fewer incidences of negative mood and engagement and more neutral mood and engagement levels when travelling on PTS.

### 3.3. Mapping Data

A detailed body of qualitative DCM observations was gained from mapping on the PTS ambulances and in the care settings which the participants were recruited from. In normal care environment maps, this observation data would be relayed in full to the care staff for each individual that was mapped in order to effect change in that person's care. In formal research however, the level of detail provided by DCM cannot be given the space that it requires; this dilemma is an issue that impacts on the transferability of DCM within formal research and evaluation studies, particularly for large samples. For the purposes of this study, the mapping data has been summarized into broad themes with some examples of mapping notes attached for supporting evidence.

## 4. Factors That Had a Positive Impact on Mood and Engagement

### 4.1. Staff Engagement

“P7's well-being (positive ME) was increased and maintained by staff through reminiscence. Many instances were seen on both journeys where communication by staff brought P7 back from a B into high potential categories and well-being. Good evidence to support recommendation on proactive engagement.”

“P8 also benefited from the engagement in conversations by staff-communication was sustained for 15 minutes and brought him back from a B into high BCC's with +3 ME. Very good example of service.”

### 4.2. Regular, Trained Escort and Driver

“P2 was greeted by PTS staff, assisted onto ambulance and strapped in. Same staff every week so escort and driver know them very well. Very bumpy ride but no-one seemed to mind due to the conversations on the ambulance. P2 said when they got off that the journey didn't upset them. No negative mood at all, 100% positive!”

“P3 has MS. Most of journey was flat experience for her despite the efforts of escort who welcomed her on, chatted all the time to P's and made them laugh. Escort very engaged with involving them all, asking if they were cold, and so forth. 3 personal enhancers (PE's) (Personal Enhancers (PE's) are markers of positive person work and demonstrate the level to which care workers attempt to uphold personhood (Brooker & Surr, 2005).) were recorded in 25 minutes from the escort.”

“P13 is also blind and wheelchair bound; accompanied by careworker (CW) from nursing home. Long first journey for P13; CW sat talking to her trying to engage her in conversation—made her laugh at points—explained reasons for ambulance stopping, and so forth. For the return journey P13 was not engaged and became withdrawn at one point. CW should have been more proactive in improving her experience of the journey as she is blind and needed someone else to be her eyes. Again, provides support for the need for quality of escort and content of care.”

### 4.3. Engagement with Quiet or Less Independent Passengers

“P5 showed anxiety, holding book and turning it over. Driver engaged her about the book (PE) and her ME value went from a −1 to a +3 within 10 minutes. Return journey was less positive—10 minutes prior to pick-up P5 became quite anxious folding tissues, and so forth. For the first 35 minutes of the return journey P5 was in negative ME, showing clear signs of anxiety—nobody spoke to her until the mapper intervened and brought her back into positive ME. This happened twice; therefore P5 could have been in 50 minutes of continuous negative ME.”

“Lots of positive interaction on return journey for P10, with the crew maintaining communication about her old job and so forth Her ME value would drop to a +1 when not being spoken to but would rise to a +3 when conversation was resumed; again emphasizes the quality of escorts!”.

## 5. Factors That Had a Negative Impact on Mood and Engagement

### 5.1. Lack of Information about Travel Schedules

“No designated PTS team or escort on journey. P1 waited 45 minutes for ambulance after coming out of appt. No time of arrival for ambulance given to him. P1 complained about waiting for ambulance and the discomfort of sitting in wheelchair too long.”

### 5.2. Lack of Planning for Toilet Visits

“P4 had wetting accident in clinic while waiting for ambulance. Staff took him to the toilet and gave him spare clothes to change into (not his own) and put his own into a plastic carrier bag. P4 looked very embarrassed and kept saying “I'm sorry”. When on the ambulance he kept touching his trousers and seemed distressed by this. The incident sent him into negative mood and could easily have been avoided.”

“When P6 got on the ambulance for the return journey she asked to go to the toilet—had to be taken off and back into the clinic, which delayed the transport leaving and increased anxiety for other passengers.”

## 6. Discussion

This study found that Dementia Care Mapping can be used successfully as a practice development tool, to monitor individual patient's mood and behaviour during transport to and from clinics on PTS ambulances. It provides a rich description of the journey and can raise awareness in services interested in improving the patient experience for this significant part of their care. However, this study has shown the feasibility of using DCM in research to be problematic for a number of reasons. A major limitation relates to the fact that DCM has historically been used primarily as a practice development tool, not as an outcome measure. Therefore, the very act of using DCM within a setting will undoubtedly influence the environment and confound observations. Also a measure should not be used as both the intervention and the outcome within a study. The data that DCM produces for individual participants can be very detailed and this does not lend itself well to large-scale projects involving a large sample of participants. Given the limitations of feasibility that this study has found when using the tool for research purposes, DCM would not be recommended as the sole intervention or outcome measure for future research, particularly large scale studies.

The results from this study however do suggest that the type of support provided by PTS staff is of great importance to the patient experience. The positive benefits of having designated staff teams with an escort were seen clearly in the Behaviour Category Codes (BCC's) profile for the two groups and in the mapping data; however, quantitative analysis of the ME values did not support a significant difference. In response to the research question, “What are the observed behaviours and well-being of service users with dementia during a typical patient journey on PTS?,” the BCC profiles showed that the range of observed behaviours differed across the two groups while the ME values did not show significant benefits. ME values record two dimensions of patients' experience: their level of mood state (positive and negative) with the extent to which they are engaged with their activity, the people and environment around them. Therefore, ME values can reflect either of those dimensions and this could potentially skew the data recorded. 

However, the study showed that Behaviour Category Codes were most positive in the escort group with no low potential categories seen and only 24% of time spent in B (borderline), which is a medium potential category, compared to 30% in the no escort group. The BCC of “borderline” which describes being passively engaged (e.g., watching) is what would be expected on transport such as a bus or ambulance, as most passengers will spend time looking out of the window, so this was not an area of concern. Our data has shown that the mood of patients, particularly vulnerable groups such as dementia patients, is adversely affected when they are not responded to or appear to be ignored by staff. This cannot always be avoided as a lone driver cannot be expected to compromise the safety of his or her passengers and must concentrate on negotiating the traffic, assisting other passengers on and off the transport, and responding to passengers as best they can. We have shown the benefit of having an escort present on the ambulance to provide dedicated support to the passengers.

Furthermore, the wide range of behaviours seen in the escort group was all positive and the variety seen would be reflective of a formal care environment such as a day hospital. Creative activities such as “Expressive,” “Going back”, and “Intellectual” are all high potential categories and were facilitated by the presence of familiar escorts who knew the passengers well and encouraged sing-alongs, discussions about the daily news and stories about their lives. The participants and others on the transport responded well to this type of stimulation and interaction and most reported enjoying their regular trips out. 

In response to the third research question “How does having designated PTS staff teams, with an escort, affect the well-being of people with dementia in comparison to generic PTS staff?”, the results have shown fewer incidences of negative levels of mood and engagement and a wide range of positive behaviours in the escorted groups. Travelling on PTS can be a positive experience for people with dementia if they have regular staff to travel with and an escort who is aware of and experienced in person-centred care. Many participants expressed their enjoyment of getting out of the house and seeing familiar faces which was not an expected outcome of the study. Yet for the socially isolated these trips provide regular opportunities to get out of the house and meet up with familiar faces to talk and share feelings which enhance people's quality of life. The findings show that any physical discomfort arising from PTS journeys for older people with dementia can be counterbalanced by good quality care and support from the PTS staff team. The length of journey times and the provision of escorts could also be seen to extend the therapeutic environment beyond the formal clinic or hospital setting, and further research could explore whether the duration of therapeutic intervention in this way has an impact on patient outcomes.

### 6.1. Limitations of Study

Subject reactivity is one limitation of this study which cannot be ruled out as the mapper's presence will have had an effect on the PTS surroundings, and possibly the behaviour of a staff. Other confounding variables could have been the number and behaviour of other patients travelling on the transport and the health of the participants on the day of mapping. Another limitation of this study was the small sample size which could create type 2 error, meaning the results cannot be relied upon or generalised across the wider population but can indicate trends and patterns for future research.

### 6.2. Recommendations

This study found that DCM8 can be used successfully, as a practice development tool, to map mood and behaviour during transport to and from clinics for individual patients with dementia. It provides a very detailed and rich description of journey events and can be recommended as one tool which should be considered for raising awareness by teams interested in improving the patient experience for this significant portion of a “clinic day.” However, as previously discussed, DCM would not be recommended as the only observational method or outcome measure for future research, particularly in large scale studies, as the data produced by the tool can be too unwieldy and difficult to generalise across populations. 

The importance of staff trained in person-centred care for people with dementia was supported by this study. The mapping results reported in this study could form the basis of normative data that describes a set of normal rhythms in behaviour for patients with dementia when travelling on PTS. This can also be used in future research. Staff in clinics should give attention to the positive contribution they can make to their clients' experience by preparing them for journeys by that ensuring opportunities to use toilets are provided at appropriate times. Staff in clinics should also give attention to the positive contribution they can make to their clients' experience of journeys by providing information about waiting times and journey schedules. 

In conclusion, escorts appear to improve the experience of patients with dementia during journeys to and from clinics, and the cost-effectiveness of this extension of the therapeutic environment should be the subject of further research. Larger studies that can overcome the barriers to research in this important but under-resourced area, and which use methods that are more suitable than DCM, may help to clarify these findings and identify ways to reduce stress and promote safety and well-being for patients with dementia during these necessary, but often long and arduous journeys.

## Figures and Tables

**Table 1:** Behaviour category codes (BCC) index.

Behaviour category codes (BCC)		
High potential categories		

A	Articulation	Interacting with others verbally or otherwise
D	Doing for self	Self care
E	Expressive	Expressive or creative activities
F	Food	Eating or drinking
G	Going back	Reminiscence and life review
I	Intellectual	Prioritising the use of intellectual abilities
J	Joints	Exercise or physical sport
K	Kum and go	Walking, standing or moving independently
L	Leisure	Leisure, fun and recreational activities
N	Nod (Land of)	Sleeping, dozing
O	Objects	Displaying attachment to or relating to inanimate objects
P	Physical	Receiving practical, physical or personal care
R	Religion	Engaging in a religious activity
S	Sexual expression	Sexual expression, flirting
T	Timalation	Direct engagement of the senses
V	Vocational	Work or work-like activity
X	Excretion	Episodes related to excretion
Y	Yourself	Interaction in the absence of any observable other
Z	Zero option	Fits none of existing categories

Moderate potential categories		

B	Borderline	Being engaged but passively (watching)

Low potential categories		

C	Cool	Being disengaged, withdrawn
U	Unresponded to	Attempting to communicate without receiving a response
W	Withstanding	Repetitive self-stimulation of a sustained nature

**Table 2:** Mood and engagement values.

Mood	ME Value	Engagement
Very happy, cheerful. very high positive mood.	+5	Very absorbed, deeply engrossed/engaged.

Content, happy, relaxed. considerable positive mood.	+3	Concentrating but distractible. considerable engagement.

Neutral. absence of over signs of positive or negative mood.	+1	Alert and focussed on surroundings. Brief or intermittent engagement.

Small signs of negative mood.	−1	Withdrawn and out of contact

Considerable signs of negative mood.	−3	

Very distressed. very great signs of negative mood.	−5	

**Table 3:** Participant and journey information.

N	Age	Gender	Level of dementia	Service type	Designated PTS team	PTS escort	Journey time (one way)
1	80	M	Moderate	Outpatients	No	No	35
2	78	F	Moderate	Day hospital	Yes	No	15
3	61	F	Moderate	Day hospital	Yes	Yes	40
4	71	M	Moderate	Day hospital	Yes	Yes	35
5	64	F	Moderate	Day hospital	Yes	Yes	65
6	73	F	Mild	Day hospital	Yes	Yes	25
7	79	M	Severe	Day hospital	Yes	Yes	75
8	64	M	Mild to moderate	Day hospital	Yes	Yes	25
9	85	M	Mild to moderate	Day hospital	Yes	Yes	50
10	82	F	Severe	Day hospital	Yes	Yes	20
11	74	F	Moderate	Day hospital	No	No	15
12	90	F	Moderate	Day hospital	No	No	35
13	70	F	Severe	Outpatients	No	No	55
